# Mechanisms and Countermeasures for Muscle Atrophy in Microgravity

**DOI:** 10.3390/cells13242120

**Published:** 2024-12-20

**Authors:** Yizhou Liu, Xiaojian Cao, Qiuzhi Zhou, Chunchu Deng, Yujie Yang, Danxia Huang, Hongmei Luo, Song Zhang, Yajie Li, Jia Xu, Hong Chen

**Affiliations:** 1 Department of Rehabilitation, Tongji Hospital, Tongji Medical College, Huazhong University of Science and Technology, Wuhan 430030, China; l20241021@foxmail.com (Y.L.); zephyrus@hust.edu.cn (X.C.); 2023tj0332@hust.edu.cn (Q.Z.); deng_c@tjh.tjmu.edu.cn (C.D.); m202276342@hust.edu.cn (Y.Y.); d202282137@hust.edu.cn (D.H.); luohongmei@hust.edu.cn (H.L.); d202081852@hust.edu.cn (S.Z.); 2018tj5463@hust.edu.cn (Y.L.); jiaxuz0604@hust.edu.cn (J.X.); 2 Stem Cell Research Center, Tongji Hospital, Tongji Medical College, Huazhong University of Science and Technology, Wuhan 430030, China

**Keywords:** spaceflight, microgravity, skeleton muscle, muscle regeneration, muscle atrophy

## Abstract

Previous studies have revealed that muscle atrophy emerges as a significant challenge faced by astronauts during prolonged missions in space. A loss in muscle mass results in a weakening of skeletal muscle strength and function, which will not only contribute to a decline in overall physical performance but also elevate the risk of various age-related diseases. Skeletal muscle atrophy in the microgravity environment is thought to be associated with changes in energy metabolism, protein metabolism, calcium ion homeostasis, myostatin levels, and apoptosis. Modulating some pathways could be a promising approach to mitigating muscle atrophy in the microgravity environment. This review serves as a comprehensive summary of research on the impact of microgravity on skeletal muscle, with the aim of providing insights into its pathogenesis and the development of effective treatments.

## 1. Introduction

Spaceflight presents astronauts with a multitude of risks, including muscle atrophy, cardiovascular dysregulation, central nervous system impairments, elevated cancer risk, and bone loss. These formidable challenges significantly impede the progress of the aerospace industry [1,2]. Among them, muscle atrophy stands out as a paramount pathological challenge afflicting astronauts during their orbital missions [3]. Extended periods of spaceflight can lead to a reduction of approximately 30% in muscle mass, concomitant with a decline in muscle strength [4]. Despite daily exercise during mission tasks, it remains insufficient to fully prevent the onset of muscle atrophy [5]. Simultaneously, muscle atrophy tends to deteriorate progressively with extended durations in orbit, and there is a notable absence of effective treatments for this condition [6]. Previous studies have shown that microgravity can affect the cellular biological processes of muscles [7]. However, the specific molecular mechanism behind this still requires further in-depth exploration. In addition, due to long-term disuse of skeletal muscles, a series of physiological processes such as cellular calcium metabolism disorders, energy metabolism, protein metabolism, myostatin, and apoptosis may also play a role in the process of atrophy.

Microgravity alters the energy sources of muscles, typically leading to changes in glycolysis and fatty acid oxidation processes. These shifts in energy supply directly affect various biological processes in skeletal muscles, potentially causing impaired muscle regeneration and transitions in muscle fiber types [8,9,10]. Consequently, the ability of muscles to adapt to microgravity environments diminishes.

Additionally, disruptions in protein metabolism directly impact the morphology and function of muscle fibers. Pathways such as AKT/mTOR, the ubiquitin–proteasome pathway (UPP), and endoplasmic reticulum (ER) stress significantly influence muscle regeneration and exacerbate skeletal muscle atrophy [11,12,13]. Understanding these mechanisms can help refine and optimize dietary strategies for astronauts.

Skeletal muscle, as a mechanosensitive tissue, relies on the functional state of mechanosensitive channels for its growth, particularly in a microgravity environment [3]. Changes in intracellular and extracellular calcium ion concentrations play a critical role in cell growth and metabolism. Microgravity alters the functionality of calcium ion channels, such as ryanodine receptors (RyRs) and TRP pathways, thereby disrupting the normal physiological activities of skeletal muscle cells [14,15]. Investigating calcium ion-related pathways and mechanisms is crucial for developing effective drugs to combat skeletal muscle atrophy.

Moreover, myostatin, a negative regulator that directly acts on skeletal muscles, influences muscle metabolism and hinders muscle regeneration, thereby worsening muscle atrophy in the microgravity environment [16,17,18]. Exploring its role and intervention strategies under microgravity conditions could contribute to maintaining muscle mass.

Finally, apoptosis is another critical mechanism underlying muscle atrophy in microgravity environments [19,20]. Microgravity may promote the expression and activity of apoptosis-related genes, such as P53 and caspase-3, thereby enhancing skeletal muscle cell apoptosis [21,22]. Researching ways to regulate apoptotic pathways will be instrumental in protecting muscle cells and mitigating the progression of muscle atrophy.

Currently, the precise mechanism underlying skeletal muscle atrophy in a microgravity environment remains elusive. This paper serves as a comprehensive summary of research on the impact of microgravity on skeletal muscle, aiming to offer insights into investigating its pathogenesis and developing effective treatments. Such endeavors are pivotal for the sustainable, healthy, and accelerated advancement of human aerospace endeavors.

## 2. Microgravity Environment Affects Process of Skeletal Muscle Regeneration

**Figure 1:**
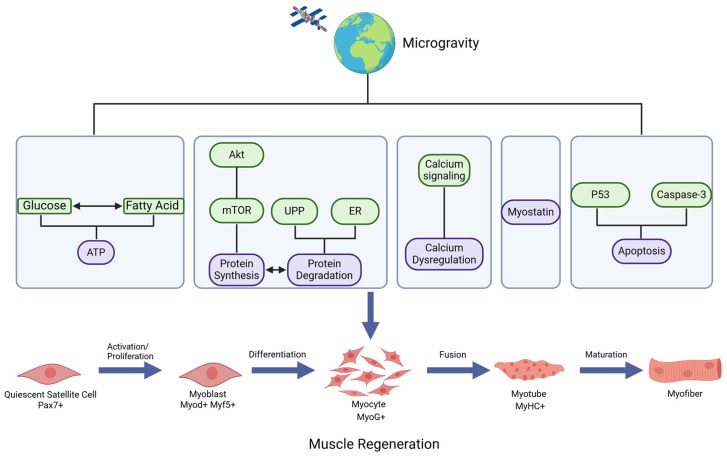
The factors affecting skeletal muscle atrophy in the microgravity environment. Skeletal muscle atrophy in the microgravity environment may result from a combination of factors, including disruptions in cellular calcium metabolism, alterations in energy and protein metabolism, increased myostatin levels, and enhanced apoptosis. (Created in https://BioRender.com, accessed on 9 December 2024).

Skeletal muscle regeneration is a sophisticated and dynamic process [23]. Within this process, muscle satellite cells (MuSCs) play a critical role in muscle regeneration progress [24]. MuSCs remain in a quiescent state in healthy and uninjured muscle, located between the myofiber sarcolemma and the basal lamina, with a specific expression of Pax7 [25]. In the context of exercise, microgravity, or disease, quiescent MuSCs quickly become active and undergo swift proliferation [26]. The activated MuSCs re-enter the cell cycle and generate proliferating myoblasts that express myogenic transcription factors like MYF5 and MYOD [27]. Subsequently, these myoblasts undergo differentiation and fuse together to form myotubes, which ultimately mature into myofibers, contributing to the repair of damaged skeletal muscles (Figure 1) [28,29]. Recent studies have revealed that microgravity can induce muscle atrophy by disrupting the process of muscle regeneration [16,30,31,32,33]. Some research indicates that muscle atrophy induced by microgravity may be attributed to the depletion of skeletal muscle stem cells. Extended exposure to microgravity not only leads to satellite cell death but also induces alterations in myostatin expression levels [16]. In particular, in vitro studies have demonstrated that myostatin inhibits myoblast proliferation and MuSC self-renewal by suppressing MyoD expression, thus leading to the inhibition of myogenesis [34]. Tohru Hosoyama and colleagues conducted a study in which induced pluripotent stem cell-derived MuSCs were cultured under a microgravity environment for 2 weeks. This microgravity environment not only resulted in reduced Pax7^+^ expression but also led to a decline in the expression levels of tumor necrosis factor receptor-associated factor 6 (TRAF6) and the subsequent phosphorylation of its downstream molecule, extracellular-related kinase (ERK), within skeletal muscle stem cell spheres. Consequently, the effects of microgravity seem to detrimentally affect Pax7 expression in MuSCs, potentially by impeding the TRAF6/ERK pathway, consequently disrupting the maintenance of the skeletal muscle stem cell pool [31]. Microgravity can also disrupt the process of muscle regeneration by impacting myotube fusion. Zhang ZK and colleagues cultured C2C12 cells, which are a subclone of the mouse myoblast cell line, in a microgravity environment for 7 days. During the differentiation process, it was observed that microgravity caused a significant inhibition of myotube fusion in C2C12 cells. This was evidenced by a much lower fusion index in the microgravity group when compared to the control group on day 7. Their findings led to the identification of a long non-coding RNA (lncRNA), referred to as “mechanical unloading-induced muscle atrophy-related lncRNA”, which acted as an anabolic regulator capable of preventing the development of muscle atrophy [35]. Some results have indicated that microgravity can enhance the proliferation of myoblasts while simultaneously causing myoblast dedifferentiation and ultimately affecting myotube fusion. In the simulated microgravity environment, the density of C2C12 cells increases, along with heightened levels of the myoblast marker Myf5, while the expression of Myog decreases. In this situation, the development of myotubes is hindered. The decrease in Ca^2+^ levels is linked to this procedure [32]. Numerous factors can contribute to inconsistent research conclusions in this field. Firstly, variations in the devices employed to simulate a microgravity environment in experiments, including 2D clinostats, 3D clinostats, random position machines (RPMs), and rotary cell culture systems (RCCSs), can lead to divergent results. Secondly, within the real space environment, unlike in simulated microgravity environments, there are other factors, such as radiation, that can also impact cell growth [36]. Lastly, variations in the chosen intervention times for experiments can also contribute to differences in research outcomes. Skeletal muscle atrophy in a microgravity environment may be influenced by multiple factors, and a comprehensive study of its pathogenesis can offer novel insights for future treatments for this disease.

## 3. The Mechanism of Muscle Atrophy Induced by Microgravity

### 3.1. The Alteration of Skeletal Muscle Energy Metabolism Pathways in the Microgravity Environment

Skeletal muscle fibers can be classified as fast-twitch fibers (type 1) and slow-twitch fibers (type 2) based on their contraction speed [37]. Slow muscles, which rely on aerobic metabolism, have a high density of capillaries and oxidative enzymes, efficiently oxidizing fatty acids and glucose to generate ATP. And slow muscles exhibit fatigue-resistant properties [38]. Fast muscles, which rely on anaerobic metabolism or glycolysis, can rapidly generate ATP, making them contract more readily and at a higher speed. Fast muscles also experience earlier fatigue compared to slow fibers because the conversion of glucose to pyruvate yields less ATP than what can be generated through the rest of the central metabolism, ultimately leading to the production of CO_2_ [39]. Studies have pointed out that the muscle fiber type gradually shifts from slow-twitch fibers to fast-twitch fibers, leading to a decrease in the number of slow-twitch fibers in the microgravity environment. However, fast-twitch fibers are more prone to atrophy and degeneration in microgravity, resulting in muscle wasting [40]. The increase in the number of fast-twitch fibers may lead to enhanced glycolysis in skeletal muscles and a relative reduction in aerobic oxidation [8]. Relevant studies have confirmed that microgravity can enhance glycolytic activity while reducing the function of key enzymes in the fatty acid oxidation pathway, particularly the activity of carnitine palmitoyltransferase 1 (CPT1) [9]. CPT1, a pivotal enzyme governing fatty acid oxidation, transfers fatty acids into mitochondria. V.P. Grichko and colleagues suggested that the apparent increase in reliance on glycogen metabolism during hindlimb unloading (HLU) could not be attributed to a diminished ability to oxidize carbohydrates or fatty acids. Instead, it is likely the consequence of substrate-level phosphorylation activation of glycolysis [10]. The reason for this phenomenon is that the enhanced glycolytic process leads to an increased production of acetyl-CoA. When the intracellular concentration of acetyl-CoA is high, it promotes the synthesis of malonyl-CoA. Furthermore, malonyl-CoA is a recognized inhibitor of CPT1, which plays a crucial role in fatty acid oxidation [41]. Ultimately, malonyl-CoA inhibits the activity of CPT1, reducing the entry of fatty acids into the mitochondria, thereby decreasing the capacity for fatty acid oxidation [8,10]. Fatty acid oxidation plays a vital role in both muscle regeneration and energy supply [42]. A reduction in fatty acid oxidation leads to insufficient energy supply for MuSCs, thereby limiting their proliferation and differentiation processes, ultimately exacerbating muscle atrophy [43]. However, this conclusion is controversial. Recent studies have suggested that microgravity promotes muscle lipid synthesis and fatty acid metabolism [22,44]. Soochi Kim and colleagues observed that after 7 days of culture on a muscle-on-a-chip platform in a real microgravity environment, skeletal muscle regeneration was impaired, accompanied by an increased expression of pathways related to fatty acid metabolism. This highlights the significant role of fatty acid metabolism in skeletal muscle atrophy in a microgravity environment [22].

It is evident that the changes in skeletal muscle metabolic patterns caused by microgravity environments require further in-depth investigation. Studying the primary energy pathways of skeletal muscle and the interactions between glucose and lipid metabolism through biological engineering, animal models, and cell experiments in a real microgravity environment will provide valuable insights for developing optimized dietary strategies for astronauts in the future (Figure 1).

### 3.2. Microgravity Can Lead to Changes in the Synthesis and Breakdown of Muscle Proteins

Muscle atrophy in the microgravity environment is a multifaceted process involving alterations in both protein synthesis and degradation [45]. The prevailing theory suggests that muscle atrophy in rat models of immobilization or HLU is mainly caused by reductions in protein synthesis [46,47]. The cellular mechanisms responsible for the decline in protein synthesis following unloading remain incompletely understood. Recent research has highlighted the crucial role of the activation of the serine/threonine kinase (AKT) and the mammalian Target of Rapamycin (mTOR) in governing the growth of adult skeletal muscle in response to increased loading [48,49]. AKT plays a pivotal role in protein synthesis regulation by activating the mTOR signaling pathway. Through this mechanism, AKT acts as a key mediator in balancing energy availability and protein production, which is critical for myoblast proliferation and maintenance [50]. The mTOR pathway increases protein synthesis and is involved in skeletal muscle hypertrophy induced by mechanical overload [49,51]. In rodent models subjected to immobilization and HLU, studies have reported decreases in the activation of Akt/mTOR in muscles like the soleus and medial gastrocnemius [48]. During HLU, a decline in protein synthesis is generally observed in all muscles [52]. The reduction in Akt/mTOR activation leads to decreased protein synthesis in C2C12 myoblasts, which in turn disrupts myotube formation and results in impaired skeletal muscle regeneration [53]. Mi-Ock Baek and colleagues also pointed out that simulated microgravity enhances the expression of PLD2, leading to the dissociation of mSIN1 from the mTORC2 signaling complex, which reduces Akt phosphorylation and ultimately inhibits the growth and differentiation of C2C12 cells [11]. However, certain research indicates that the augmentation in protein degradation also plays a role in the atrophy response triggered by disuse [54,55]. Recent research has indicated that the ubiquitin–proteasome pathway (UPP) is regarded as one of the most important pathways for protein degradation [56]. One study has shown that protein degradation in skeletal muscle in a microgravity environment is associated with the UPP [12]. l-carnitine may serve as an effective treatment for muscle atrophy in the microgravity environment by inhibiting the UPP [55]. Inhibiting the ubiquitin–proteasome pathway (UPP) reduces protein degradation, which is beneficial for myotube formation [57]. The alterations in protein homeostasis induced by microgravity may also be associated with the function of the endoplasmic reticulum (ER) [13]. The ER serves various functions as a site for protein synthesis, folding, modification, and transport [58]. Zeinab Ibrahim and colleagues explored the role of ER stress in muscle atrophy. After three weeks of HLU, they observed higher expression levels of ER stress markers in the HLU group compared to the control mouse groups. The administration of 4-PBA, a comprehensive ER stress inhibitor, partly mitigated ER stress and led to a partial restoration of muscle mass and strength in the HLU mice [13]. Moderate endoplasmic reticulum (ER) stress can activate the unfolded protein response (UPR), which helps reduce apoptosis in muscle stem cells [59]. Additionally, ER stress can promote the proliferation of muscle stem cells [60]. However, excessive ER stress may impede the differentiation of muscle stem cells [61]. These findings suggest that ER stress plays a role in muscle atrophy in an HLU environment.

Skeletal muscle atrophy in a microgravity environment may be associated with reduced protein synthesis due to the AKT/mTOR pathway as well as increased protein degradation mediated by ER stress and the UPP (Figure 1). Investigating the mechanisms of protein metabolism in skeletal muscle regeneration, along with the mechanisms underlying muscle atrophy, could help reverse the effects of reduced mechanical load on muscle function.

### 3.3. Calcium Dysregulation in Microgravity Environments

Calcium homeostasis appears to play a crucial role in regulating the response to microgravity exposure [62]. The regulation of calcium homeostasis involves a network of sensors, receptors, and channels, primarily activated by mechanical stresses [63]. Different cell types rely on mechanical signals to trigger suitable physiological responses. Through the activity of calcium channels, cells can convert mechanical stimuli into biological responses [64]. In the development and adaptability of skeletal muscle, calcium signaling plays an essential role [65]. Calcium signaling pathways can regulate the cell cycle, thereby influencing both the proliferation and differentiation of myoblasts [66]. Calzia and colleagues noted that C2C12 cells exposed to a simulated microgravity environment experienced a significant decrease in intracellular free calcium ion concentration, and this was related to ryanodine receptors (RyRs) [32]. RyRs, critical for the release of calcium from intracellular reservoirs, have been associated with exposure to simulated microgravity [14]. RyRs are linked to both mitochondrial and ER membranes and have the capability to rapidly trigger an elevation in intracellular calcium levels [67]. In a separate study, Benavides Damm T and colleagues suggested that microgravity can cause C2C12 cells to remain in the G2/M phase of the cell cycle by inhibiting the TRPC1-mediated reduction in calcium influx. As a result, the deceleration in cell proliferation hampers the terminal differentiation of myoblasts [15]. The function of TRPC1 channels has been strongly associated with cell proliferation in different types of cells, and they are the main channels through which cations divide C2C12 cells [68]. TRPC1 facilitates cell division by providing a calcium source that assists in advancing through the cell cycle. The pharmacological inhibition of TRPC1 prevents cells from completing the process of cell division [69]. TRPC1 expression peaks during the G1 phase but declines during the G2/M phase. Moreover, myoblasts exposed to a microgravity environment exhibited slower cell proliferation, which is a consequence of a delay in their transition from the G2/M phase to the G1 phase of the cell cycle. This delay is in line with the reduced expression of TRPC1. The study also pointed out that inhibiting calcium influx through TRP channels with SKF-96365 caused myoblasts to progress into the G2/M phase of the cell cycle and delayed their proliferation [15]. A decrease in TRPC1-mediated calcium influx appears to be an essential event contributing to muscle atrophy induced by microgravity.

In the microgravity environment, muscle atrophy may occur due to impaired skeletal muscle regeneration, which results from the suppression of calcium release through RyRs and reduced calcium influx via the TRP pathway (Figure 1). Further research on how changes in mechanical load affect calcium channels and the upstream and downstream signaling pathways could provide valuable insights for preventing and treating skeletal muscle atrophy in microgravity environments.

### 3.4. Microgravity Promotes the Expression of Myostatin

Myostatin, also recognized as Growth Differentiation Factor 8 (GDF-8), is a protein secreted from the Transforming Growth Factor-β (TGF-β) family [70]. Myostatin transcript and/or protein expression has been demonstrated to be modulated by diverse physiological and pathological conditions affecting muscle mass [71]. These include situations such as muscle atrophy, heart infarction, muscle unloading, HIV infection, exposure to microgravity, chemical-induced muscle damage, muscle regeneration, and muscle reloading [72]. Myostatin is widely acknowledged as a potent inhibitor of skeletal muscle growth [73]. Remarkable increases in skeletal muscle mass have been linked to the disruption or inhibition of myostatin gene expression, mainly due to muscle fiber hyperplasia and hypertrophy [74]. Myostatin not only impedes muscle cell growth but also hampers differentiation. This multifaceted function contributes significantly to its regulatory role in skeletal muscle development and maintenance [75]. The suppression of myostatin results in an upregulation of MyoG and MyoD expression. Additionally, the suppression of myostatin also led to an accelerated growth rate of myoblasts, evident through an increased cell proliferation index, coupled with heightened expression levels of cyclin D1 and cyclin E [76]. Research indicates a potential link between escalated myostatin mRNA and protein levels in skeletal muscle and the depletion of skeletal muscle mass observed during spaceflight [16,17,18]. Umberto Tarantino and colleagues suggested that satellite cells respond to simulated microgravity by increasing the expression of myostatin [16]. In another study, researchers proposed that exposure to microgravity could trigger the upregulation of myostatin, contributing to the loss of muscle mass [17]. And blocking myostatin signaling with a myostatin-specific peptide can partially alleviate muscle atrophy and dysfunction caused by hindlimb unloading [77]. Se-Jin Lee and colleagues demonstrated that mice deficient in the myostatin gene (Mstn(−/−) exhibited an approximately two-fold increase in muscle mass across their bodies compared to wild-type mice. Moreover, a lack of myostatin has demonstrated promise in counteracting muscle atrophy triggered by microgravity [78]. The signaling of myostatin involves a complex that includes type II and type I receptors. This signaling cascade initiates upon the binding of these ligands to the activin type II receptors ACVR2 and ACVR2B. The decoy receptor ACVR2B/Fc, which incorporates the extracellular, ligand-binding domain of ACVR2B, fused with an immunoglobulin Fc domain, has exhibited potent inhibition of myostatin signaling, which led to substantial muscle growth [79]. The overall muscle weights of mice exposed to a microgravity environment and treated with ACVR2B/Fc were preserved. This underscores the effectiveness of inhibiting this signaling pathway in enhancing muscle growth in a microgravity environment [78]. These findings illustrate that while myostatin plays a crucial role in maintaining skeletal muscle mass, certain studies have suggested that myostatin may not be essential for atrophy caused by microgravity. Research has shown that Mstn(−/−) mice experienced a greater reduction in body and quadriceps femoris mass compared to wild-type mice. Moreover, during HLU, Mstn(−/−) mice experienced a reduction of approximately 33% in the mass of the extensor digitorum longus (EDL) muscle [80]. This implies that the lack of myostatin in Mstn(−/−) mice may make them more vulnerable to atrophy induced by unloading.

These observations have caused speculation as to whether increased myostatin expression could play a role in sarcopenia observed in connection with spaceflight (Figure 1). The products of the myostatin genes could potentially serve as targets for the development of countermeasures to prevent muscle loss during spaceflights.

### 3.5. Microgravity Alters the Apoptosis Process of Skeletal Muscle Cells

Apoptosis is a tightly regulated process of programmed cell death that plays a crucial role in maintaining tissue homeostasis and is involved in various biological processes [81]. The process of cell apoptosis involves complex biological mechanisms, including the interaction between pro-apoptotic and anti-apoptotic factors [82]. Studies have shown that microgravity can regulate the apoptosis process of various cell types, including cancer cells, embryonic stem cells, osteocytes, and chondrocytes [19,81,83]. Similarly, microgravity environments can enhance the apoptosis process in skeletal muscle [22,30]. Radugina and colleagues observed that mouse muscle tissue in a real microgravity environment contained a higher number of apoptotic cell nuclei compared to the control group. They also suggested that c-myc and c-jun may be involved in the apoptosis of skeletal muscle cells in the microgravity environment [30]. The apoptotic regulator protein caspase-3 is also believed to be associated with muscle atrophy in microgravity [21,84]. Caspase-3 is a cysteine-aspartic protease, and its main function is to execute the apoptotic process by cleaving and inactivating various intracellular proteins, particularly poly (ADP-ribose) polymerase (PARP) [81]. After 2 weeks of hindlimb unloading in mice, the activity of caspase-3 in their soleus muscle was significantly increased [21]. Furthermore, Lee and colleagues suggest that the effect of oenothera odorata root extract may prevent apoptosis in C2C12 cells under a microgravity environment by activating HSP70 and inhibiting caspase activity [85]. Studies have also indicated that microgravity environments can upregulate the expressions of AEN and PHLDA3, enhancing the apoptosis process in skeletal muscle [22]. AEN and PHLDA3 are believed to be involved in the P53-mediated apoptosis process [86]. p53 is a critical tumor suppressor that triggers the apoptosis process in response to DNA damage or cellular stress [87]. On the contrary, other studies have also suggested that microgravity does not necessarily enhance the apoptosis process in skeletal muscle [31,88]. Lorenzo Sanesi and colleagues have pointed out that hindlimb unloading increases the expression of apoptosis-related genes, such as P53, in the cortical bone of mice. However, this phenomenon has not been observed in skeletal muscle [88].

In summary, muscle atrophy in a microgravity environment may be associated with multiple apoptosis pathways (Figure 1). Identifying the pathways of apoptosis in microgravity environments and utilizing effective methods to regulate the apoptosis process could serve as a promising research direction in the future.

## 4. Strategies for Muscle Atrophy Prevention in Microgravity

### 4.1. Torpor Might Play a Role in Muscle Atrophy in Microgravity

The phenomenon of natural hibernation is a captivating yet mysterious physiological process in which energy conservation is achieved by reducing the metabolic rate, body core temperature, and behavioral activity during difficult seasonal conditions [89]. Hibernation employs a complex regulatory system to coordinate a homeostatic state of reduced metabolism, lower body temperature, and decreased activity in response to environmental challenges [90]. Hibernation, by significantly reducing energy consumption, minimizes the need to carry substantial amounts of food and fluids for life-support systems [91]. This conservation of payload capacity benefits spaceflight. In contrast to an active human, an astronaut in a metabolically quiescent state demands notably reduced amounts of supplements and living space [92]. For instance, if an astronaut’s metabolism is suppressed through hibernation, their energy intake could be reduced by as much as 75% [93]. Furthermore, inducing a state of hibernation in astronauts might aid in alleviating the physiological impact of weightlessness on muscles and bones, in addition to the expected advantages in resource conservation [94]. The inherent physiological characteristics of torpor might contribute to maintaining the integrity of organs and tissues. Hibernating mammals display minimal or no reduction in bone mass and muscle strength even after extended periods of inactivity and decreased mechanical loading during torpor [95]. After 6–8 months of hibernation, bears show no significant loss in bone mass and experience less muscle mass and strength loss than would typically be expected after such an extended period of physical inactivity [96]. In the majority of disuse atrophy models, there is a notable increase in the proportion of fast glycolytic muscle fibers [97]. Conversely, research on hibernating animals has revealed only a minor decrease in oxidative type I fibers and a consistent maintenance of fiber ratio during the winter season [98]. Additionally, during hibernation, the metabolic rate slows down, reducing the animal’s energy requirements and subsequently decreasing the activity of glycolysis [99,100]. Through the study of torpor, a theoretical foundation can be established for simulating or inducing hibernation. Nowadays, it is becoming increasingly feasible to induce a torpor-like state in humans through pharmacological or other intervention methods [101]. Torpor is a short-term state of metabolic slowdown, usually lasting from a few hours to a day, with relatively mild physiological changes. In contrast, hibernation is a long-term state of metabolic reduction, typically lasting several weeks to months, often accompanied by more significant drops in body temperature and a greater reduction in metabolic activity [102]. Dimethylsulfoxide, pentobarbital, Neuropeptide Y1, and other pharmacological treatments have been examined for their ability to induce metabolic depression, but they have not been able to replicate the full range of responses observed in natural torpor [103,104,105]. Significant progress has been made in understanding the anatomical and functional connections that control torpor within the central nervous system [90]. Tohru M. Takahashi and colleagues have shown that the chemo-genetic excitation of a specific population of neurons in the mouse hypothalamus with a particular genetic identity and spatial location induces an extremely long-lasting state of regulated hypometabolism that is similar to torpor [106]. Furthermore, ultrasound stimulation of the hypothalamic preoptic area can induce a torpor-like state in mice, characterized by hypothermia and metabolic suppression [107]. Studies have shown that the number of MUSCs in ground squirrels is regulated by P21 during different stages of hibernation [108]. P21 plays a critical role in cell cycle regulation by halting progression in the G1 phase, thereby modulating cell proliferation [109]. Although muscle regeneration is delayed, MUSCs are still able to perform their functions without causing fibrosis in the muscle tissue [110].

**Figure 2:**
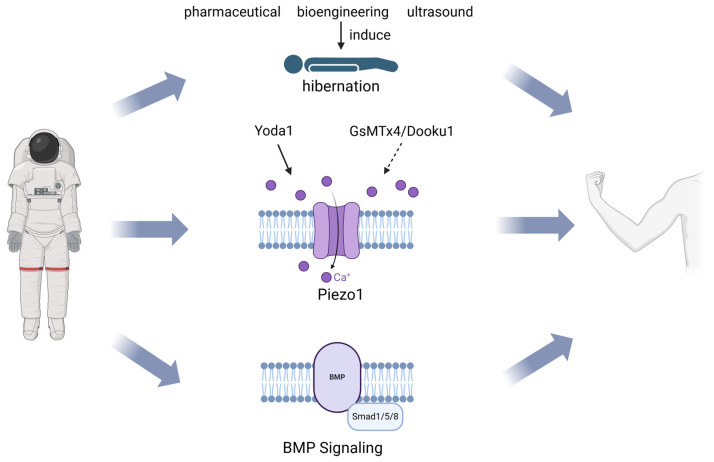
Strategies for treating skeletal muscle atrophy in a microgravity environment. Inducing hibernation in astronauts and modulating the functional status of the Piezo1 or BMP signaling pathways are potential strategies for treating skeletal muscle atrophy in a microgravity environment. (Created in https://BioRender.com, accessed on 9 December 2024).

Applying methods such as pharmaceuticals, bioengineering, and ultrasound to induce a torpid state in humans could help mitigate the adverse effects of long-term space travel and offer potential solutions for future deep-space exploration (Figure 2).

### 4.2. Modulating Piezo1 Activity Is a Potential Strategy for Muscle Atrophy in Microgravity

The stability of the internal environment of tissues and cells is not only regulated by biological and chemical signals within the body but also influenced by their physical environment, including other factors such as tension, stress, and gravity [111]. As a result, mechanical force plays a crucial role in governing cell growth [112]. Skeletal muscle is a tissue that is highly responsive to mechanical forces, and these forces have a profound impact on its structure, mass, and function [49]. Nowadays, it is widely accepted that mechanical stimulation is an essential factor in promoting the regeneration of skeletal muscle [113,114]. Mechanosensitive (MS) channels are ion channels that can be activated by mechanical forces. These channels play a role as mechanical transducers in different physiological processes, such as hearing, touch and pain perception, osmotic regulation, and blood pressure regulation [115]. The Piezo1 channel is a highly mechanically activated, non-selective cation channel that can sense mechanical stimuli and convert them into physiological signals in various tissues, including skeletal muscles [116]. The Piezo1 channel primarily transports Ca^2+^ but is also permeable to other cations, including Na^+^ and K^+^ [117]. Piezo1 influences critical signaling pathways, including MAPK/ERK and YAP/TAZ, which are essential for muscle cell proliferation and differentiation [118]. Piezo1 is present in quiescent MuSCs at the initial stage. As MuSCs are activated, the expression of Piezo1 gradually decreases. If Piezo1 is deleted, there is a noticeable reduction in the quantity of Pax7^+^/Myod^−^MuSCs, implying that Piezo1 is essential for sustaining the skeletal muscle stem cell population [119,120]. Kotaro Hirano and co-workers revealed that during the cell division process of MuSCs, Piezo1 is clearly localized in the midbody. Furthermore, MuSCs with Piezo1 deficiency exhibit an abnormal chromosomal structure, which contributes to the decrease in the number of MuSCs. This phenotype contributes to the impairment of Piezo1-Rho signaling during myogenesis [120]. Another study has shown that Piezo1 deficiency leads to the compensatory upregulation of the T-type voltage-gated calcium channel, resulting in an increased influx of Ca^2+^, which strongly induces the expression of NOX4 through classical protein kinase C (cPKC). The increased expression of NOX4 in Piezo1-deficient MuSCs leads to elevated levels of reactive oxygen species (ROS) and DNA damage. This, in turn, results in P53-dependent cellular senescence and cell death [119]. Recent studies have suggested that a decrease in the expression of Piezo1 results in a reduction in myoblast fusion, ultimately leading to a decline in the formation of myotubes—a crucial process in muscle development and regeneration [119,121]. The relocation of phosphatidylserine, advanced by phospholipid flippases, to the cell surface is a critical step for initiating Piezo1 activation. The activation of Piezo1 stimulates the entry of calcium, enabling the assembly of actomyosin through RhoA/ROCK, thereby regulating cell fusion and guiding the extension of myotubes in a polarized mode. Furthermore, the activation of Piezo1 using Yoda1 can enhance myoblast differentiation, promote myotube fusion and elongation, and induce significant calcium transients in myotubes without triggering an increase in the proliferation of MuSCs [122]. The microgravity environment can disrupt the equilibrium of intracellular tension, resulting in the formation of an irregular cytoskeleton. This significantly impacts the modulation of cell growth, movement, and survival [123].

Studying the functional state of Piezo1 in the microgravity environment and regulating its activity using the Piezo1-specific activator Yoda1 and inhibitor GsMTx4 or Dooku1 will be beneficial in combating muscle atrophy induced by microgravity (Figure 2).

### 4.3. BMP Signaling Contributes to the Regeneration of Skeletal Muscle

Studies utilizing transgenic and knockout mice, as well as observations from animals and humans with naturally occurring mutations linked to BMPs, have provided evidence for the crucial functions of BMP signaling in different developmental processes, including bone, muscle, and neuron development. This signaling pathway is integral to the regulation and coordination of these developmental processes [124,125,126]. Signal transduction investigations indicate that Smad1, Smad5, and Smad8 act as direct downstream molecules of BMP receptors, playing pivotal roles in the transmission and mediation of BMP signals. These Smads are critical components of BMP signaling, mediating cellular responses to BMP ligands, which are crucial for diverse developmental processes, including those related to bone, muscle, and neuron development [127]. Without injury, the application of Bmp7 prompts heightened Smad1/5 phosphorylation, fostering muscle fiber hypertrophy. This hypertrophy is mediated through the Insulin-like Growth Factor (IGF) and AKT/mTOR signaling pathways [128,129]. Research has demonstrated that BMPs are also involved in the regulation of skeletal muscle regeneration [130,131]. During the early stages of the activation process, MuSCs are responsive to BMP signals. BMP signals prevent activated MuSCs and proliferating myoblasts from exiting the cell cycle, leading to a larger population of MuSC descendants. In contrast, the inhibition of BMP signaling contributes to a decrease in the density of myoblasts and an increase in myotube fusion [131]. The excessive activation of BMP signaling, achieved by releasing the miR-26a blockade on Smad1/Smad4/Id3, ultimately caused a delay in the muscle regeneration process [132]. Similarly, excessive activation of BMP signaling through the Acvr1 receptor could lead to reduced muscle size during regeneration and heightened fibrosis after muscle injury, emphasizing the crucial need for the precise regulation of BMP levels during the muscle regeneration process [133]. Umberto Tarantino and colleagues conducted research on how MuSCs respond to simulated microgravity environments. In the very early phases of the simulated microgravity environment, the MuSCs exhibited greater activity compared to those subjected to the normal gravity regime. This increased activity was evident through the higher number of myotubes and a significant increase in the expression of BMP-2 in all experimental groups [16].

These studies have provided insights that allow us to propose a potential molecular mechanism in the response to simulated microgravity environments, confirming the significance of BMP signaling in the physiopathogenesis of muscle tissue (Figure 2).

## 5. Conclusions

Skeletal muscle, as the most mechanically sensitive tissue, exhibits a high degree of responsiveness to mechanical stimuli [134]. The skeletal muscle system has evolved to play a crucial role in maintaining stability and body posture under the constant influence of gravity loads [135]. Prolonged periods of skeletal muscle inactivity or mechanical unloading (immobilization, HLU, bed rest, spaceflight and reduced steps) can lead to a substantial loss in musculoskeletal mass, size, and strength. These conditions ultimately result in muscle atrophy [136]. Recent research suggests that skeletal muscle atrophy in a microgravity environment is the result of the combined action of various mechanisms [137]. Moreover, the negative impact of a microgravity environment on the regeneration of skeletal muscle significantly exacerbates muscle atrophy [7]. The absence of mechanical loading in microgravity reduces the stimulation of muscle cells, impairing their proliferation and differentiation [138]. However, the precise molecular mechanism of muscle atrophy in a microgravity environment remains elusive. Further research into the mechanism and signaling pathways involved will provide us with a better understanding of microgravity-induced muscle atrophy and improve our ability to prevent and treat this condition. Exploring the biological mechanisms behind hibernation in animals, investigating the function of the mechanically sensitive channel Piezo1, and understanding the BMP signaling pathway could provide valuable insights into potential strategies for mitigating muscle atrophy in a microgravity environment. This research may shed light on innovative approaches and interventions aimed at preserving muscle mass and function during long-duration spaceflights and addressing the challenges associated with muscle atrophy in such conditions.

## Data Availability

No new data were created or analyzed in this study.
